# Swept-source multimode fiber imaging

**DOI:** 10.1038/s41598-023-34062-6

**Published:** 2023-05-18

**Authors:** Benjamin Lochocki, Aleksandra Ivanina, Akje Bandhoe, Johannes F. de Boer, Lyubov V. Amitonova

**Affiliations:** 1 Nanoscale Imaging and Metrology, Advanced Research Center for Nanolithography (ARCNL), Science Park 106, 1098 XG Amsterdam, The Netherlands; 2 Department of Physics and Astronomy, LaserLaB, Vrije Universiteit Amsterdam, de Boelelaan 1081, 1081 HV Amsterdam, The Netherlands

**Keywords:** Optics and photonics, Imaging and sensing, Microscopy, Optical physics

## Abstract

High-resolution compressive imaging via a flexible multimode fiber is demonstrated using a swept-laser source and wavelength dependent speckle illumination. An in-house built swept-source allowing for independent control of bandwidth and scanning range is used to explore and demonstrate a mechanically scan-free approach for high-resolution imaging through an ultrathin and flexible fiber probe. The computational image reconstruction is shown by utilizing a narrow sweeping bandwidth of $< 10$ nm while acquisition time is decreased by 95% compared to conventional raster scanning endoscopy. Demonstrated narrow-band illumination in the visible spectrum is vital for the detection of fluorescence biomarkers in neuroimaging applications. The proposed approach yields device simplicity and flexibility for minimally invasive endoscopy.

## Introduction

In the development of smaller and minimal invasive endoscopes, ultra-thin fibers play a vital role. Many fiber-based endoscopy configurations have been proposed: bundles^1–3^, fibers with miniaturized optics^4,5^, multi-core fibers (MCF)^6,7^ and multi-mode fibers (MMF)^8–12^. MCF or MMF provide imaging in hard-to-reach places by creating spatially resolved light patterns at the distal fiber end and collecting the signal from the sample. Usually, a monochromatic beam is sequentially scanned across the fiber input facet generating independent illumination patterns at the sample plane. The patterns illuminate the sample and the signal is collected and measured via a “bucket” detector. The single-pixel configuration allows for the exploration of compressive sensing algorithms, by using a sub-Nyquist set of patterns and the computational super-resolution reconstruction of the object^13–20^.

However, the crucial component in all those configurations is the need for a light scanning or wavefront shaping device to create desired illumination patterns at the fiber output. Commonly, either galvanometric mirrors^17^, a spatial light modulator (SLM)^11,21^ or a digital micromirror device (DMD)^22,23^ are used. Choudhury et al. reported on using a single mode fiber (SMF) to sequentially illuminate the cores of a MCF, by mounting the proximal end of the MCF onto a computer controlled stage^7^. Spatial raster scanning or spatial wavefront shaping have many drawbacks including complexity of the setup, low speed, and mechanical instability. Here we explore a new way to generate dynamic illumination patterns for MMF imaging.

The wavelength of light can be used as an additional degree of freedom to control the spatial profile at the fiber output. Different wavelengths (optical frequencies) have different speed while propagating through the waveguide and therefore have different phase delays between guided modes of the MMF on the output^24,25^. This property enables unique wavelength dependent speckle patterns at the output facet. Single-pixel imaging via compressive sensing and wavelength dependent scattering in $TiO_{2}$ layer has been recently demonstrated^26^. A similar concept using an all-fiber setup was proposed by Kubota et al. in^27^. However, no data on probe’s flexibility and imaging robustness have been reported. Moreover, in these works, near infrared (NIR) illumination was used, which makes the demonstrated systems not suitable for future applications in fluorescent imaging, since most fluorophores operate in the visible wavelength range.

Here we demonstrate an ultra-thin and flexible probe for high resolution fiber imaging in the visible domain using a custom-made swept laser source and a combined singlemode-multimode probe. Different samples have been visualised and investigated. Robustness of the imaging approach was tested by doing calibration measurements prior imaging with subsequent fiber movements mimicking real application endoscopic movement. The proposed approach does not rely on any spatial raster scanning or wavefront shaping system and can potentially provide super-resolution endoscopic imaging.

## Methods

### Experimental setup

**Figure 1:**
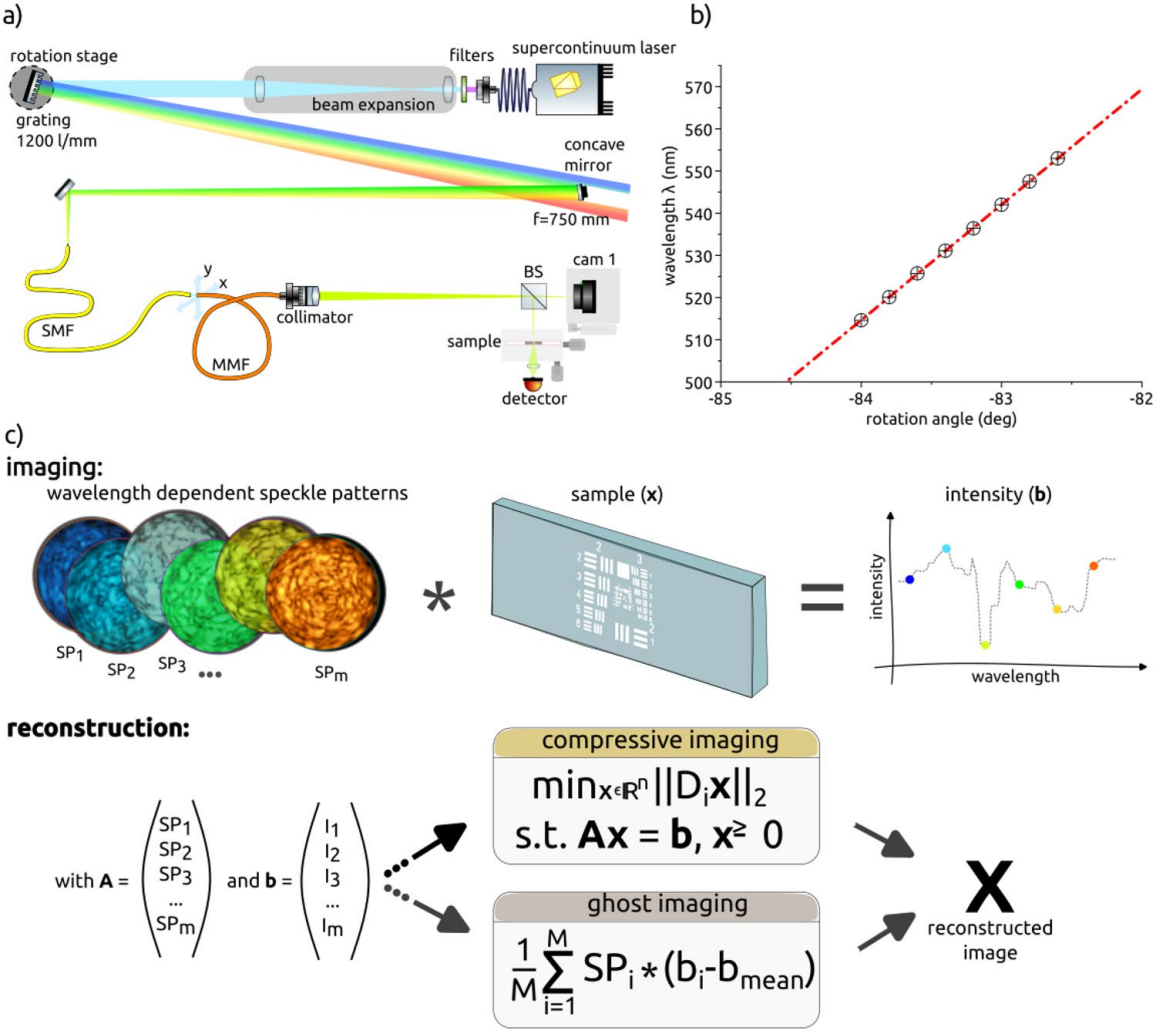
(**a**) Schematic of the experimental setup. A diffraction grating is illuminated by the collimated VIS spectrum of a supercontinuum laser. Light, which is dispersed into separate wavelengths is coupled to a single-mode fiber, which serves as a pinhole selecting a single wavelength. The single-mode fiber is connected to the MMF. Wavelength dependent speckle patterns generated in the MMF illuminate the sample and the total response is collected by a bucket detector. (**b**) Wavelength at the MMF output as a function of the rotation angle of the grating: experimental data (circles) and linear fit (red line). (**c**) The main principle of swept-source compressive fiber imaging. Wavelength dependent speckle patterns “illumination matrix” (**A**) and the corresponding signal from the sample (vector **b**) are measured and feed either into the ghost imaging algorithm or into a regularization algorithm to find the solution **x**, which is the image of the sample.

A swept-source, based on a super-continuum laser (Leukos Rock 400, 4W) and a custom-made monochromator was built allowing for an access to multiple degrees of freedom, such as scanning speed, step size, spectral bandwidth of each illumination pattern and the total bandwidth. The experimental setup is presented in Fig. 1a. The near-infrared wavelength range of the pump light was filtered out via two low-pass filters (Thorlabs, DMLP900) and a band-pass filter (Thorlabs FESH0750) such that only visible (VIS) light in the range from 400 to 750 nm was transmitted further. The beam was expanded by a factor of 8.33, using two achromatic lenses (f = 30 mm and 250 mm). The expanded VIS beam hits a reflective grating (Thorlabs GR50-1205 rules, 1200 l/mm with 500 nm blaze) mounted on a high-resolution rotational stage (Physics Instruments V-611.998061, connected to a C-891.130300 PIMag controller). The stage can be rotated in $1~\mu$rad step sizes (equivalent to 0.000057$^\circ$) allowing for precise wavelength selection. The first order fanned-out light reflected from the grating was focused via a concave mirror (Thorlabs, CM254-750-E02, f = 750 mm) onto the the tip of a single-mode fiber (SMF, Thorlabs, P1-460Y-FC-1, core diameter  $4~\mu$m), which acts as a pinhole. Based on the components, the theoretical diffraction limited resolution of the monochromator is 0.02 nm. The narrow wavelength bandwidth used for illumination leads to relatively low intensities of the pump light. High-power tunable laser sources could be used for future applications, as our proof-of-principle experiments demonstrate that a relatively small scanning range allows for imaging.

The SMF output was sturdily mounted on a 2D *x*-*y* stage opposite to the input facet of a sturdily mounted multimode fiber (MMF, Thorlabs M42L01, core diameter 50 $\mu$m, length: 1 *m*). The *x*-*y* stage allowed to vary light coupling parameters. We investigated different coupling positions, from the center to the edge of the fiber and conclude that a slight off-center position give the best speckle patterns, in terms of homogeneous distribution of speckles. The results can be seen in Suppl. Fig. S1. The close distance of less than $50 \mu$m between SMF and MMF ensures that most of the light from the SMF is coupled into the MMF even for off-center positions.

To calibrate the custom made monochromator, a spectrometer (Ocean Insight, OceanHDX, max. resolution 0.7 nm) was placed at the distal end of the SMF. The output spectrum measurements at different rotation angles were taken. The data was linearly fitted, as can be seen in Fig. 1b. Since the monochromatic output wavelength is linear related to the rotational position of the grating, a rotation of $1^\circ$ equals a wavelength shift of around 27.5 nm. That means, a rotation of $0.001^\circ$ (which is within the resolution limits of the rotation stage), allows a wavelength shift of 0.027 nm (which is matching the resolution of the monochromator) and similar to the resolution achieved by Redding et al. using a MM fiber for low-cost spectroscopy^28^.

A large-beam achromatic fiber collimator with an adjustable focus (Thorlabs, C40FC-A) or a fiberport (Thorlabs PAF2-A4A) projected the light onto the sample with a magnification of $M = 11.1$ or $M = 44$, respectively. The magnified diffraction limit has been calculated as $M\cdot \lambda / (2\text {NA})$, where $\lambda = 500$ nm is the smallest wavelength used. A R70:T30 beam splitter (Thorlabs BS022) directed the reflected beam towards the sample and the transmitted beam towards a camera (Basler a2a1920-160um) to record the speckle patterns. An avalanche photodiode (APD, Thorlabs APD440A2) in combination with a collimating lens or a camera (Basler acA1300-200um) was used to measure the total intensity transmitted through the sample. The camera mounted close to the back side of the sample operates as a large area detector increasing measurement sensitivity. During the processing step, the camera image was integrated, and results in one intensity value per related speckle pattern, as truly for a single pixel detector. The intensities of the speckle patterns vary with wavelength, as can be seen in Suppl. Fig. S2d. These wavelength-dependent intensity variations have been taken into account because both the illumination matrix **A** acquired during the calibration and the intensity vector **b** recorded during the measurements are linearly dependent on the corresponding intensities.

The grating on the stage was rotated over a scanning range of one degree. At each stage position, the software waits 2 ms before a camera acquisition was triggered. The binary transparent samples, “two dots”, “hand-written zero digit” and “three bars” were in-house made photolithographically objects etched onto a standard-size aluminum sputtered microscope slide. As a “hand-written zero digit” sample, we use a random digit from the standard *MNIST* database. The “resolution target bars” sample was part of a negative resolution target (Thorlabs R2L2S1N). During experiment, the samples were mounted in a *x*-*y* sample holder (Thorlabs, XYF1/M), which was additionally mounted on a linear stage to obtain *z*-direction displacement.

### Imaging protocol

The imaging and reconstruction principle is depicted in Fig. 1c. For each wavelength, a unique speckle pattern was passively created via the light scrambling in the MMF. The examples of speckle patterns for 15 different wavelengths between 514 to 541.5 nm in steps of 1.83 nm are shown in Suppl. Fig. S2a. The cross-correlation graph and correlation histogram for all the experimentally measured speckle patterns are shown in Suppl. Fig. S2b and c, respectively. For each color coded speckle pattern, projected on a sample, the camera acquired an image and the detector behind the sample recorded the total transmitted intensity. Background measurements were not taken.

The acquired stack of speckle images and related intensities data were loaded and processed in Matlab 2022a (MathWorks). The speckle images were pre-processed by extracting their circular region of interest and by zero padding the remaining corners. For image reconstruction, the two different methods Ghost Imaging (GI) and Compressive Imaging (CI) were used. Ghost imaging, as in the single pixel imaging approach reconstructs an object, *O*(*x*, *y*), as the weighted sum of speckle pattern ($SP_i$) with the coefficients calculated as subtraction of the mean intensity ($b_{mean}$) from the speckle related intensity ($b_i$) measurement^29^:

1 $$\begin{aligned} O(x,y) = \frac{1}{M} \sum _{i=1}^{M} (b_i - b_{mean}) \times SP_i(x,y), \end{aligned}$$

where *M* is the number of measurements. Thus, each bucket measurement $b_i$ is the overlap between the object and the illumination pattern. The GI can be seen as a vector projection of the object transmission function over M different random vectors $SP_i$. Achieving a good contrast, requires in practice $M>> N^{2}$ patterns, which is not given here, therefore the GI reconstruction could be very poor or not given at all^30^.

Compressive Imaging reconstruct a sample from a series of measurements by finding solutions to underdetermined linear systems using the sparsity constrain. Among several popular regularization algorithms available we chose to use TVAL3^31^. TVAL3 often offers a fairly good solution without any specific precondition due its adaption to non-sparse sample, compared to other denoising algorithms which work best when the sample is sparse. Furthermore, the TVAL3 algorithms is preferably used due to its superiority in computational speed. The isotropic $TV+$ model was chosen for TVAL3: $min_{x\in {\mathbb {R}}^{n}} \sum \limits _{i}\left\| D_{i}x \right\| _{2}$, s.t. $Ax = b$, $x\ge 0$; with **A** the illumination matrix (the reshaped speckle patterns), **b** the measured intensity per speckle pattern and **x** the sample and with $D_{i}x$ the discrete gradient of *x* at pixel *i* as described in detail in^31^. The variables can be split by introducing $y_{i} = D_{i}x$, and the TV model is now equivalent to: $min_{y_{i},x} \sum \limits _{i} \left\| y_{i} \right\| _{p}$, s.t. $Ax = b$ and $D_{i}x = y_{i}$, with $p = 1$ or 2. The following non-default parameters were set to: maxit = 1500, TVnorm = 1, nonneg = true and isreal = true. TVAL3 allows to set an initial guess as starting reference for its reconstruction routine which might positively influence the result. In this study, the computed results of GI were used as initial guess. However, we did not notice any significant advantage of setting this parameter compared to its default setting of a zero matrix.

For comparison of CI to conventional raster scan imaging, we establish as metric the compression rate ($CR = (N \times N)/{M}$), which is a measure to indicated the decrease in acquisition time in CI. CR was defined by the $N \times N$ pixel size of a reconstructed image, divided by the amount of speckle patterns (*M*) used to illuminated the sample. A point-by-point raster scan image needs $N \times N$ acquisitions while the CI image uses only a fraction ${M} /(N \times N)$ but retains or even enhances resolution beyond the diffraction limit^13,15,16^. The higher CR, the higher the compression, the shorter the acquisition time.

## Results and discussion

**Figure 2:**
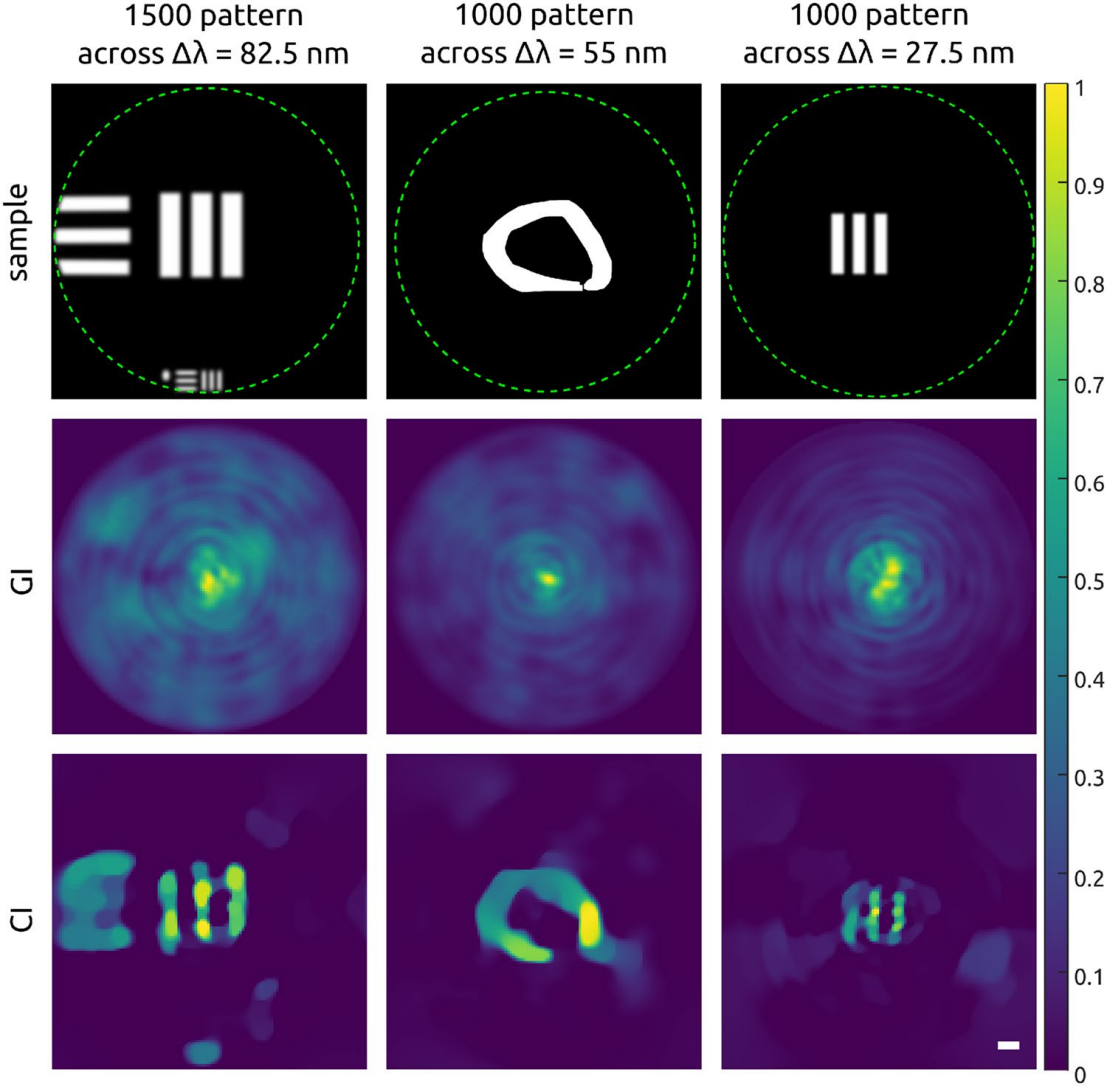
Swept-source multimode fiber imaging results for different samples (first row) using GI (middle row) and CI (bottom row) reconstruction algorithms. Resulting image size was $N^2 = 128 \times 128$. The resolution target (first column) was imaged using 1500 speckle patterns, a scanning step size of 0.055 nm and a bandwidth of $\Delta \lambda = 82.5~$nm, resulting in CR = 10.9. “Hand-written zero digit” sample (second column) was imaged using 1000 speckle patterns, a scanning step size of 0.055 nm and $\Delta \lambda = 55\,$nm, resulting in CR = 16.4. “Three bars” sample (third column) was imaged using 1000 speckle patterns, a scanning step size of 0.027 nm and a bandwidth of $\Delta \lambda = 27.5~$nm, resulting in CR = 16.4. Scale bar: 3 $\times$ diff. limit with a diff. limit of 12.6 $\mu m$.

In the first set of experiments, different samples, like “bars”, the “hand-written zero digit” and part of a “resolution target” were imaged by the proposed swept-source fiber imaging approach. The results are shown in Fig. 2. The wavelength scanning bandwidth was set to $\Delta \lambda = 55$ nm and 82.5 nm, and a total of $M = 1000$ and 1500 speckle patterns were used with a scanning step size of 0.055 nm. The overall acquisition time depends on the number of patterns projected onto the sample, the exposure time of the camera used to record the speckle images and the rotation stage settling time. For 1000 speckle patterns, an exposure time of 100 ms and a settling time of 2 ms, the overall imaging time was around 102 s.

The results of GI, shown in the second row of Fig. 2, are not representing the sample but rather display an accumulation of high signals at the center of the image. This is to be expected since GI relies on a high number of pattern for good reconstruction. In contrast, the obtained CI reconstructions of the “resolution target” with $N^2 = 128 \times 128$ pxl ($555 \times 555~\mu$m$^{2}$) resembling the sample with soft and blurry edges, which allows for the sufficient identification of the imaged object. The less complex “zero” digit is rendered almost perfectly albeit some minor background artefacts are visible. Similar results were obtained for the pure “three bars” sample. Overall, most segments of the samples were well reconstructed and could be identified easily via the CI-based swept-source fiber imaging.

### Factors that impact image quality

**Figure 3:**
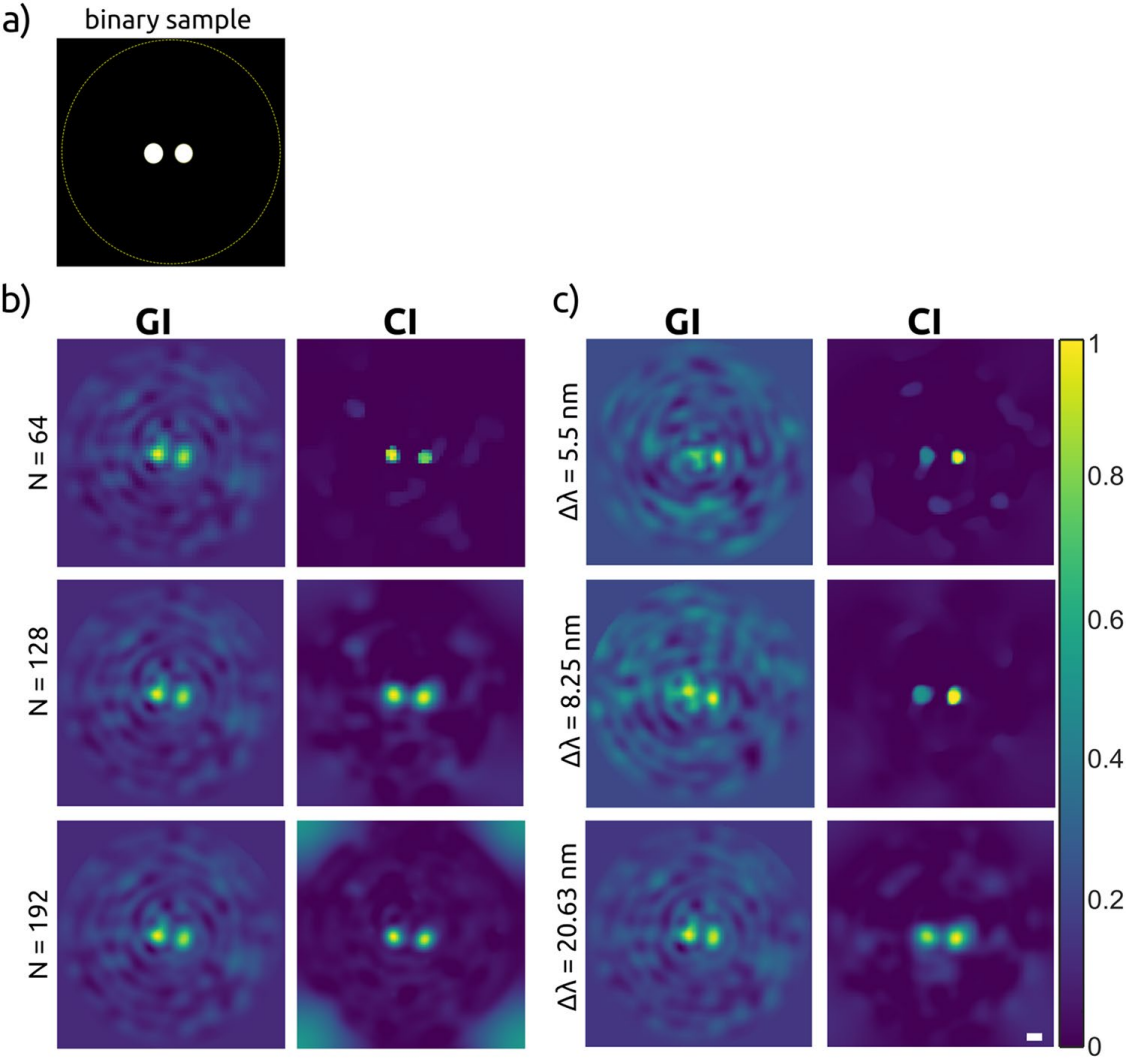
(**a**) Sample. (**b**) Swept-source multimode fiber imaging results for $\Delta \lambda = 27.5~$nm and M = 1000 patterns as a function of image size within same field of view: $64\times 64$ pxl (top row, CR = 4.1), $128\times 128$ pxl (middle row, CR = 16.4) and $192\times 192$ pxl (bottom row, CR = 36.9) using ghost imaging (first column) and compressive imaging (second column) reconstruction algorithms. c) Swept-source multimode fiber imaging results for image size of $N^2 = 128 \times 128$ pxl as a function of bandwidth (or number of speckle patterns): $\Delta \lambda = 5.5$ nm (top row, M = 200, CR = 81.9), $\Delta \lambda = 8.25~$nm (middle row, M = 300, CR = 54.6) and $\Delta \lambda = 20.63~$nm (bottom row, M = 750, CR = 21.8) for ghost imaging (first column) and compressive imaging (second column) algorithms. For simple binary objects, imaging using a small bandwidth and high CR is feasible. Scale bar: 3 $\times$ diff. limit with a diff. limit of 12.6 $\mu m$.

In the next set of experiments, the influence of the final image size on the reconstruction quality has been investigated. For that, the simple binary sample has been chosen as depicted in Fig. 3a. The holes of a diameter $45~\mu$m are separated by a center to center distance of 75 $\mu$m. We opted for a scanning range of 27.5 nm with a rotation of $1^\circ$ in 1000 steps, starting at 514 nm. The scanning bandwidth and the number of illumination patterns (M = 1000) were kept constant during the measurements.

In Fig. 3b, the reconstructed results for different N are presented. The reconstruction image size was varied from N = 64 to N = 192, resulting in different CR from 4.1 to 36.9, respectively. The first column in Fig. 3b displays the GI results, which do not depend on N. The two dots are clearly visible albeit noisy artefacts are present in the background, only the pixel grid gets finer with the increasing number of pixels.

Swept-source multimode fiber imaging using CI is shown in the second column. The visibility of the two dots is increased with the number of pixels in the reconstructed image. A better signal-to-background ratio, mainly obtained by a diminishing of the background is the result of the denoising capabilities of the TVAL3 algorithm. For $N^2 = 128 \times 128$ pxl, meaning a resolution of $4.3~\mu m$ per pixel, the holes are presented by 10 pixels, matching the size of the sample perfectly. Compared to GI, compressive imaging (with total variation denoising) increases the visibility of the two dots by dampening the background and increasing contrast by sharpening the edges of the two dots. Figure S3 in the Supplementary Information demonstrates image reconstruction quality simulated for different signal-to-noise (SNR) levels. Our simulations show that for the relatively low noise case (SNR $> 10$), good image reconstruction of a sparse sample could be achieved with the bandwidth of only 5.5 nm.

In the second set of experiments, we investigated the imaging quality as a function of bandwidth and the correspondent number of illumination patterns. The results for three different number of speckle patterns: M = 200, 300 and 750, which correspond to bandwidths of $\Delta \lambda = 5.5, 8.25$ and 20.63 nm, respectively, are presented in Fig. 3c. Since the image size of $N^2 = 128 \times 128$ pxl was kept constant, the CR value is changing (CR = 81.9, 54.6 and 21.8) based on the number of patterns used. For GI, depicted in the first column, the more patterns used the better is the result. The best results, in terms of contrast and visibility, could be obtained using CI, as shown in the second column. A bandwidth of $\Delta \lambda > 8.25$ nm, meaning only M = 300 patterns (CR = 54.6), suffices for a convincing reconstruction of the sample. This provides an ample improvement in terms of reducing the scanning bandwidth compared to the report of Shin et al.^26^ where 0.1 nm steps across 100 nm were used. Our results are in line with the well-known fact that compressive sensing depends on the sparsity of the signal: The relatively sparse object (Fig. 3c) can be reconstructed with a high compression rate, while for less sparse samples (Fig. 2), a lower CR is required.

### Imaging via a flexible probe

For real life applications it is crucial to maintain invariant speckle patterns when the fiber is moved. In the next set of experiments, we investigated the stability of the system over time and imaging performance while the fiber probe is moved.

**Figure 4:**
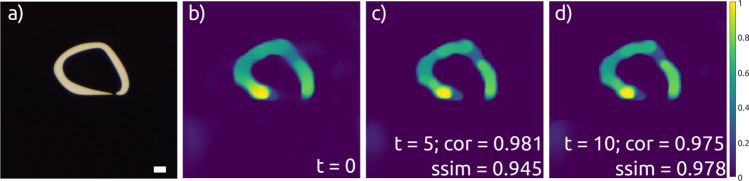
Stability over time measurements for swept-source MMF imaging using M = 1000 patterns over $\Delta \lambda = 27.5~$nm. (**a**) “Hand-written zero digit” sample. (**b**–**d**) CI reconstruction results for $N^2 = 128 \times 128$ and a CR of 16.4: illumination matrix ***A*** and intensity vector ***b*** were measured simultaneously (**b**), ***A*** and ***b***$_{5\text {min}}$ were measured with 5 min delay in between (**c**), and ***A*** and ***b***$_{10\text {min}}$ were measured with 10 min delay in between (**d**). The correlation coefficient values and structural similarity index between (**b**) (**c**) and (**b**), (**d**) are indicated. Scale bar: 3 $\times$ diff. limit with a diff. limit of 50.1 $\mu m$.

In the first experiments, we tested the reconstruction stability over time while the fiber is kept stable. The sample and the swept-source multimode fiber imaging results as a function of time in minutes are shown in Fig.  4. At zero time, the illumination matrix ***A*** consisted of M = 1000 flattened speckle patterns in the range from $\lambda _1 = 514$ to $\lambda _N = 541.5$ nm ($\Delta \lambda = 27.5$ nm), and the correspondent signal vector ***b*** were measured simultaneously. The image reconstructed by the CI algorithm is presented in Fig. 4b. It resembles, although not perfect, the complex structure of the sample with the opening and thinning-down regions. The reconstructed images have a size of $N^2 = 128 \times 128$ pxl (2203x 2203 $\mu m^{2}$). We repeated intensity measurements 5 minutes and 10 minutes later to get ***b***$_{5\text {min}}$ and ***b***$_{10\text {min}}$, respectively. Images in Fig. 4c and d show the reconstruction results for hybrid measurements: the illumination matrix ***A*** was measured at zero time with the assumption that the speckle patterns would not change over time and the signal from the sample was measured 5 min and 10 min later, respectively. The Pearson correlation coefficients (corr2) between the zero time reconstructed image and images reconstructed after delayed intensity measurements are equal to 0.98 and indicated in Fig. 4c and d. For both time stamps, the correlation values are high, indicating an excellent agreement with the original sampling. Similar results are obtained by calculating the structural similarity index (ssim). Visually, the same assessment can be concluded. Swept-source multimode fiber imaging system provide robust measurement result over time.

**Figure 5:**
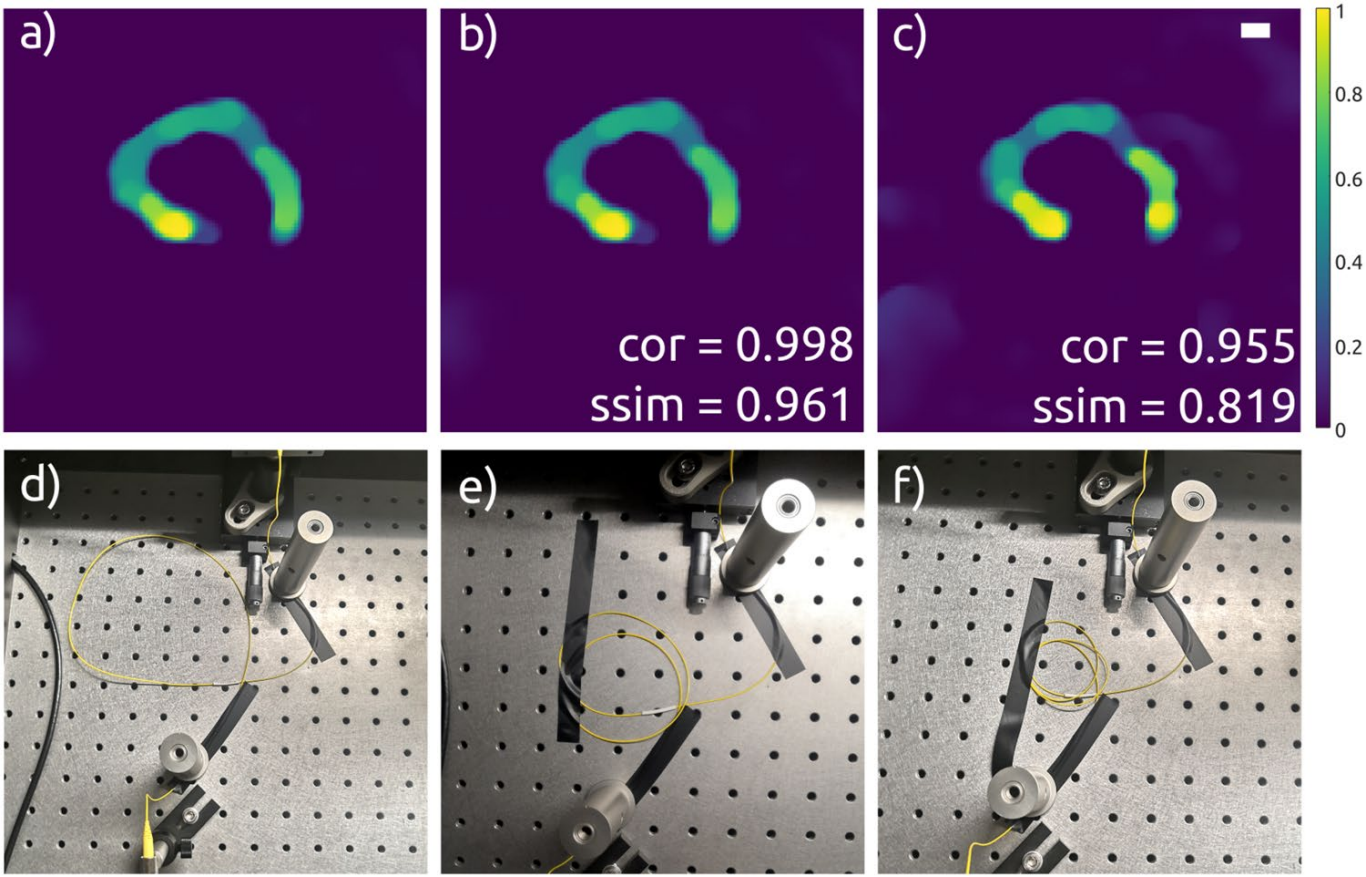
Stability measurements of the proposed swept-source MMF imaging ($\Delta \lambda = 27.5~nm$, M = 1000) during fiber movements. Image reconstruction results (**a**–**c**) and the correspondent configuration of the SMF (**d**, **e**). Pre-calibration has been done only once. Scale bar: 3 $\times$ diff. limit with a diff. limit of 50.1 $\mu m$.

In the second set of experiments, we investigated how moving and bending the single-mode part of the fiber probe would influence the imaging performance of the proposed approach. First, we measured simultaneously the illumination matrix ***A*** consisted of M = 1000 flattened speckle patterns in the range from $\lambda _1 = 514$ to $\lambda _N = 541.5$ nm ($\Delta \lambda = 27.5$ nm), and the correspondent signal vector ***b***. The image reconstructed using CI algorithm is presented in Fig. 5a. We clearly see our sample. The experimental layout of a SMF (yellow) that corresponds to the measurements is shown in Fig. 5d. Then, the SMF was coiled twice as shown in Fig. 5e and the intensity measurements were repeated. The image presented in Fig. 5b was reconstructed using the illumination matrix ***A*** measured for the first position of the SMF (as in Fig. 5d) and the intensity vector ***b***$_{2\text {loops}}$ measured for the second position of the SMF (as in Fig. 5e).

Subsequently, a third loop was added, as depicted in Fig. 5f. The image presented in Fig. 5c was reconstructed using the illumination matrix ***A*** measured for the first position of the SMF (as in Fig. 5d) and the intensity vector ***b***$_{3\text {loops}}$ measured for the third position of the SMF (as in Fig. 5f). The Pearson correlation coefficients between the image presented in Fig. 5a and the image presented in Fig. 5b was very close to 1. The correlation coefficients between the image in Fig. 5a and c was calculated to be 0.955. The correlation coefficient slightly drops with changing the SMF configuration from a single loop to three loops. Nevertheless, taking the harsh treatment and bending into account, the overall image reconstruction quality remains very good. The “zero” can be clearly distinguished from the background, although a minute increase in background noise can be noted in Fig. 5c. We conclude, that the deformation of the SMF has no major influence on the reconstruction quality even for quite extreme bending. However, it is important not to lose sight of the fact that the one meter long MMF remains the sensitive part of the setup. External influence like bending or movement will alter the light transmission through the MMF and would constantly require new calibration. To establish a robust but flexible endoscopic fiber imaging system, a shorter MMF part should be used. In addition, further ideas to generate invariant and repetitive speckle patterns (e.g., 3D printed diffractive optical elements or meta-material layers combined with swept-source illumination) could be explored.

## Conclusion

In this study, we proposed and demonstrated a swept-source fiber imaging approach. We have shown the feasibility of using wavelength dependent fiber-generated speckle patterns for fiber-based compressive imaging. We were able to reconstruct a variety of samples using a bandwidth of $\Delta \lambda < 10$ nm in the visible range. The relatively narrow VIS bandwidth could potentially be used for fluorescence imaging applications. The proposed imaging approach does not require any mechanical scanning device, which simplifies the whole illumination process compared to experimental setups with galvanometric mirrors or DMDs^12,17^. Surely, the in-house built monochromator is an over-engineered device, and can be replaced by a supercontinuum laser with an acousto-optic tunable filter or by a rapid swept source laser as commonly used in optical coherence tomography or by a tunable laser as reported lately^26,27,32^. We experimentally demonstrated that the imaging quality of the proposed approach doesn’t require stability of the fiber probe. The results open up new avenues in the field of label-free and label-based deep tissue imaging, potentially enabling *in vivo* all-fiber based endoscopic high resolution imaging.

## Supplementary Information

Supplementary Information.

## Data Availability

The datasets used and/or analysed during the current study is available from the corresponding author on reasonable request.
