# Supplementary material for: Swept-source multimode fiber imaging
Source: Sci Rep. 2023 May 18;13:8071. doi: 10.1038/s41598-023-34062-6 (PMC10195867; doi:10.1038/s41598-023-34062-6)
Supplement: Supplementary file 1 — Supplementary Information. [file 41598_2023_34062_MOESM1_ESM.pdf]

SUPPLEMENTARY INFORMATION
Swept-source multimode fiber imaging

BENJAMIN LOCHOCKI,[1,*] ALEKSANDRA IVANINA,[1] AKJE BANDHOE,[1] JOHANNES F. DE BOER,[2] AND LYUBOV V. AMITONOVA [1,2]

[1]*Advanced Research Center for Nanolithography (ARCNL), Science Park 106, 1098XG Amsterdam, The Netherlands*
[2]*Department of Physics and Astronomy, LaserLaB, Vrije Universiteit Amsterdam, 1081HV Amsterdam, The Netherlands*
[*]*lochocki@arcnl.nl*
https://arcnl.nl/research-groups/nanoscale-imaging-and-metrology

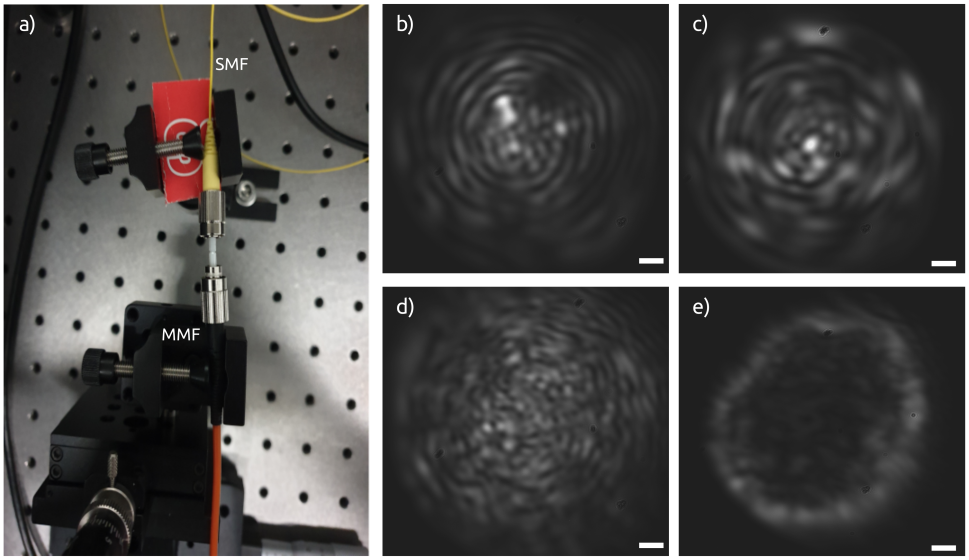

Figure S1. a) Free space fiber coupling between the single mode fiber (SMF, Thorlabs P1-460Y-FC-1) and the multimode fiber (MMF, Thorlabs M42L01). b)-e) obtained speckle patterns for $\lambda = 487.29$ nm while changing coupling position. b) and c) coupling close to the MMF center. d) Coupling slightly more off-axis, generating a homogeneous speckle pattern (preferred configuration) and d) coupling close to the core edge and hence guiding partially in the cladding. Scale bar: 200 μm.

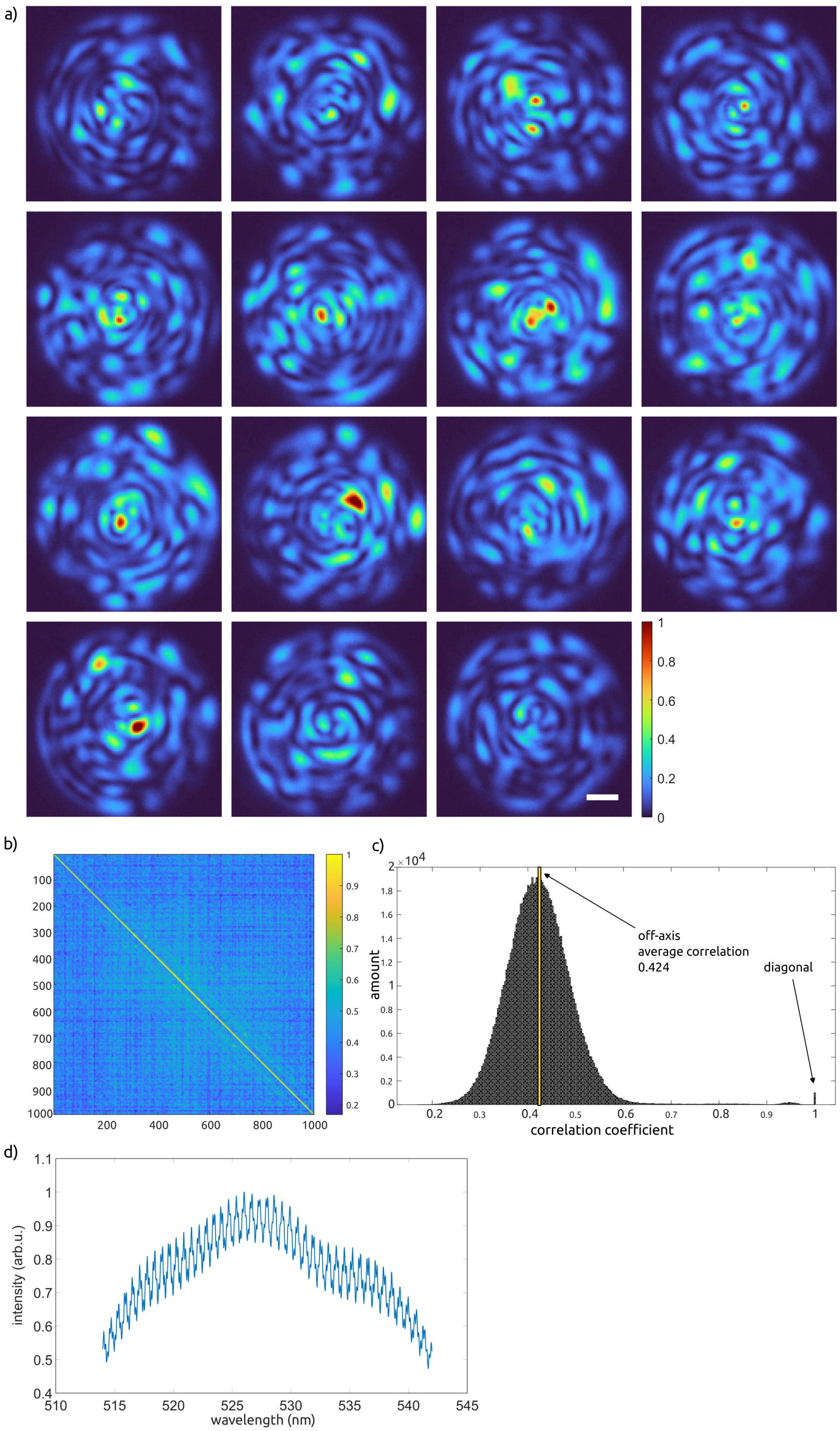

Figure S2. a) Examples of normalized speckle pattern for 15 different wavelengths between 514 to 541.5 nm in steps of 1.83 nm. The scale bar is 100 μm. b) Correlation graph and its corresponding c) correlation histogram calculated for every pair of experimentally measured speckle patterns. The off-axis average correlation of 0.42 (orange line). Please note that the off-axis correlation value is higher than expected due to the zero padding in the corners (of the circularly cropped speckle patterns). d) Normalized speckle intensity as a function of wavelength.

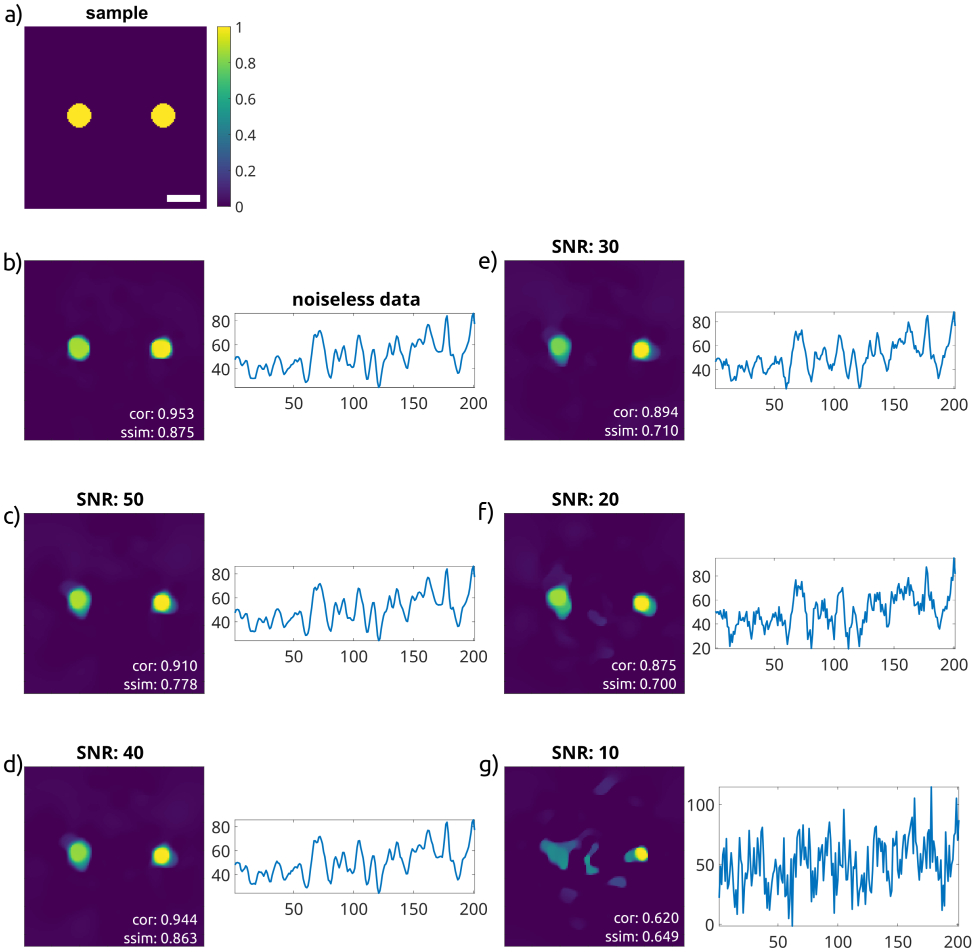

Figure S3. Swept-source fiber imaging simulation results. A set of 200 experimentally pre-recorded speckles patterns with a total scanned bandwidth of 5.5 nm is used as illumination matrix **A**. (a) The binary sample. (b-g) Reconstructed images and the simulated data (the vector **b**) for different levels of noise. The Pearson correlation coefficient and the structural similarity index relative to the original sample are indicated within each image. The diffraction limit is 12.6 μm. The scale bar is 100 μm.

Swept-source fiber imaging with different noise levels was simulated as presented in Figure S3. For the simulations, we used 200 experimentally recorded speckle patterns with a total scanning bandwidth of only 5.5 nm (a step size of 0.0275 nm) and binary sample object **x** (two dots presented in Fig. S3(a)). Each speckle has a size of $N^2 = 128 \times 128$ pxl (pixel size is 4.3 μm) corresponding to 555×555 μm field of view. The optical diffraction limit is 12.6 μm. The two dots have a diameter of 16 pxls each, which equals to 68.8 μm.

The speckle patterns (each pattern corresponds to a row in matrix **A**) were used to "illuminate" sample **x**. Illumination was simulated by the multiplication of each speckle pattern with the sample pixel-wise. The total response, which is one element in the vector **b**, was calculated by integration. The regularization algorithm, TVAL3, was fed by the illumination matrix **A** and the signal vector **b** to reconstruct the sample. The results for noiseless simulations are presented in Fig. S3(b).

To simulate the reconstruction under different signal-to-noise (SNR) conditions, we added white Gaussian noise to the simulated signal **b** by using the `awgn` function (`MATLAB`).

The SNR was defined as the difference in variance, $\Delta\sigma$, between the noiseless data **b** and generated noisy signal, $\mathbf{b}_{SNR}$.
